# Changing of HCV Clade Pattern in Iran; the Possible Means for Something Good

**DOI:** 10.5812/hepatmon.11879

**Published:** 2014-01-06

**Authors:** Muhammad Sohail Afzal, Sadia Anjum, Najam us Sahar Sadaf Zaidi

**Affiliations:** 1 Atta ur Rahman School of Applied Biosciences, National University of Science and Technology, Islamabad, Pakistan

**Keywords:** Hepatitis C, Genotype, Prevalence, Therapeutics, Iran

*Dear Editor,*

Hepatitis C virus (HCV) with around 180 million infected patients worldwide is the leading cause of mortality and morbidity (1). The prevalence of HCV in Iran is about 0.2% (2). Geographically, HCV genotype has specific distribution pattern in the world. However, in many countries it is currently observed that the genotype pattern is going to change. Changing HCV clade pattern is mainly due to world globalization, and traveling to other countries especially to the neighboring ones. 

There is an interesting and conclusive report on HCV genotype prevalence in Iran published by Jahanbakhsh Sefidi et al. (2013). The study was based on the analysis of HCV genotype prevalence from 2003-2011. The results showed that in 2003, genotype 1a was the most common (47.8 %) and decreased over time (44.9 % in 2011). It was the same for genotype 1b. HCV genotype 3a was shown to be increased over time; in 2003 its prevalence was 30.1% which was increased up to 39.6% in 2011 (3). The results also showed that genotype 3a was more prevalent in young generation demonstrating that this genotype was recently introduced to Iranian population and might be increased in the future. Geographically, Iran is surrounded largely by Pakistan, Afghanistan, Iraq, Turkey, and Kuwait. In Pakistan, more than 10 million individuals are infected with HCV and major prevalent genotype (i.e. about 50% of infections) is genotype 3a. (4). In Iraq, major prevalent HCV genotypes are 1a, 1b, and 3a (5). To our knowledge, there is no report from Afghanistan on HCV genotyping. Genotype 1b is more common in Turkish infected patients (6). In the neighboring countries of Iran, genotype 1a and 1b are common along with 3a. In Iran, the changing pattern from genotype 1 to 3a might be due to traveling between Iran-Pakistan and Iran-Iraq along the borders. Once infected individuals are located in the country, many factors are involved in spreading the new genotype of viral strain. These factors among many others may include host immune system probably not very efficient against new viral strain, intravenous drug abuse, unhealthy dentistry practices, and barbers' unawareness (7). 

The standard therapy of interferon plus ribavirin against HCV infection exhibits various efficacies on different viral genotypes. Forty six percent of patients infected with genotype 1 achieved sustained virologic response (SVR), while the SVR shown by patients infected with genotype 3 was more than 80 % (8). The changing pattern of HCV clades in Iran showed that HCV 3a might be more prevalent genotype in future. The genotype 3a revealed a very great SVR among interferon-treated patients, compared to genotype 1. Smaller SVR leads to chronicity and hepatocellular carcinoma in large number of infected individuals, while greater SVR will help in saving a lot of money and effort in dealing with infected patients. In Iran, HCV prevalence is very low, and this changing pattern to dominancy of genotype 3a may help to contain viral spread and chronicity.
